# Facilitators and Barriers to Role Adaptation in Newly Graduated Nurses in Medical-Surgical Units: A Qualitative Content Analysis Study: Short title: Role Adaptation in Newly Graduated Nurses

**DOI:** 10.31661/gmj.v15i.3910

**Published:** 2026-06-19

**Authors:** Hadi Jafarimanesh, Shahrzad Ghiyasvandian, Bret Lyman, Hossein Ghanaati, Masoumeh Zakerimoghadam

**Affiliations:** ^1^ Department of Nursing, Shazand School of Nursing, Arak University of Medical Sciences, Arak, Iran; ^2^ Department of Medical-Surgical Nursing, School of Nursing and Midwifery, Tehran University of Medical Sciences, Tehran, Iran; ^3^ Department of Medical-Surgical Nursing, Tehran University of Medical Sciences, Tehran, Iran; ^4^ Brigham Young University, 563 KMBL, Provo, UT 84602, United States of America; ^5^ Department of Radiology, School of Medicine, Imam Khomeini Hospital Complex, Tehran University of Medical Sciences, Tehran, Iran

**Keywords:** Newly Graduated Nurses, Role Adaptation, Medical-surgical Units, Qualitative Study, Facilitators, Barriers, Transition, Professional Socialization

## Abstract

**Background:**

The high turnover rate among newly graduated nurses (NGNs) is a significant concern globally, particularly in medical-surgical units (MSUs). Identifying the key facilitators and barriers to their professional role adaptation is crucial for improving retention and facilitating a smoother transition into clinical practice. This study explored facilitators and barriers to professional role adaptation among NGNs in MSUs.

**Materials and Methods:**

A qualitative study was conducted using inductive content analysis. Twenty-one NGNs with less than one year of clinical experience were purposively selected from teaching hospitals affiliated with Tehran University of Medical Sciences. Data were gathered through semi-structured in-depth interviews and analyzed using Graneheim and Lundman’s five-step approach with MAXQDA software.

**Results:**

The findings revealed that multiple interacting factors shape role adaptation at three levels: individual, interpersonal, and organizational. Facilitating factors included personal motivation, clinical preparedness, supportive colleagues, and structured organizational support. In contrast, barriers such as stress, lack of structured orientation programs, negative work culture, and excessive workload hindered adaptation.

**Conclusion:**

This study revealed that the adaptation of NGNs to professional roles in MSUs is shaped by individual, interpersonal, and organizational factors. Enhancing structured support at all three levels — through targeted educational programs, professional communication, and organizational infrastructure — can improve transition outcomes, reduce early turnover, and promote workforce retention in healthcare settings.

## Introduction

The high turnover rate among newly graduated nurses (NGNs) is a global issue and a significant concern for healthcare systems worldwide [1]. Numerous studies have examined the attrition rate of NGNs, particularly within the first year of employment. For instance, this rate has been reported to range between 12% and 50% in the United States [2], 32.7% in Iran [3], and 26.4% in South Korea [4]. The early resignation of nurses leads to multiple adverse outcomes for healthcare organizations, including a decline in the quality of nursing care, increased patient readmission rates, prolonged hospital stays, and reduced patient safety [5][6]. In addition to these care-related consequences, significant financial burdens are also imposed on healthcare systems. For example, one study estimated that a single nurse's turnover cost ranges from $82,000 to $ 88,000 [7].

One of the primary reasons for early turnover among NGNs is the difficulty in adapting to their professional role [8]. Adjustment refers to the situation in which a new nurse must adapt to the challenges of a new work environment [9]. Role adaptation is a process through which individuals utilize various cognitive, behavioral, and social strategies to manage the pressures and challenges of the workplace, gradually achieving relative stability in their new role [10]. According to Roy’s adaptation model, adaptation is a dynamic process in which individuals consciously strive to establish coherence and integration between themselves and their surrounding environment, enabling them to perform effectively within their professional context [11]. The transition period for NGNs, which involves moving from an academic setting to a clinical and professional environment, is highly challenging [12]. Kramer first introduced this transitional phase under the concept of Reality Shock [13]. In later studies, Duchscher referred to this phenomenon as Transition Shock, emphasizing that this period is marked by distinct stressors and challenges that can lead to significant psychological and occupational strain among NGNs [14]. According to the Organizational Socialization Theory, successful adaptation during the transition phase is a prerequisite for professional role stabilization, and failure to do so may result in NGNs' early withdrawal from the profession [15]. Many NGNs choose to leave the nursing profession precisely during this transitional period [16].

Therefore, identifying the factors influencing role adaptation among NGNs during this critical period is very important. Numerous studies have shown that inadequate or delayed responses to the support needs of NGNs during the adaptation process can lead to adverse outcomes, including fatigue and burnout, role conflicts, turnover intention, reduced quality of life, low job satisfaction, and actual turnover [17][18]. Previous research has identified several key factors influencing the transition period for NGNs. These include the level of professional self-confidence and physical and psychological stress [19], unrealistic expectations [20], insufficient professional knowledge and skills [21][22], heavy workload, bullying, and hierarchical abuse [23].

A comprehensive review of the literature reveals that most prior studies have been conducted in Western healthcare systems, with limited attention to how organizational and cultural characteristics in Middle Eastern contexts, such as Iran, influence role adaptation, particularly in high-demand clinical settings, such as medical-surgical units (MSUs). These units are among the most challenging environments for NGNs due to their fast-paced nature, diverse patient populations, complex care protocols, high workloads, and frequent interruptions. Such conditions impose substantial cognitive and emotional demands on nurses who are still developing their clinical judgment, technical skills, and professional confidence. The cumulative pressures can hinder effective role adaptation, intensify transition shock, and increase the likelihood of early attrition. Therefore, examining the facilitators and barriers specific to this context is essential to inform supportive strategies that enhance transition outcomes and workforce retention.

In Iran, NGNs often enter clinical settings with minimal structured orientation, limited supervisory support, and high patient care demands. The Iranian healthcare system is characterized by resource constraints, centralized management, and hierarchical communication structures, all of which may complicate the professional transition process. Additionally, cultural norms such as deference to authority, reluctance to express concerns, and limited interdisciplinary dialogue can hinder open communication and collaborative problem-solving. These systemic and cultural factors create a uniquely challenging context for role adaptation, particularly in high-acuity departments like MSUs.

Given the necessity of a deeper exploration of the factors influencing professional role adaptation among nurses and considering the critical importance of MSUs as high-traffic and high-acuity departments, this study was conducted to qualitatively explore the interacting individual, interpersonal, and organizational factors shaping this process within Iranian teaching hospitals. By offering localized insights into this transition, the findings of this research aim to inform culturally responsive educational and organizational strategies that can reduce burnout, improve satisfaction, and enhance workforce retention among NGNs.

## Materials and Methods

**Table:** Table1. Interview Questions

No.	Questions
1	During your first year as a newly graduated nurse in a medical-surgical unit, what experiences helped you adapt to your professional role? (Facilitators)
2	What challenges or barriers did you face while adapting to your professional role during this period? (Barriers)
Probing Questions	Probing questions were used to deepen the interviews, such as, "Could you elaborate on that experience?" Can you give a specific example? How did that situation influence your adaptation? What do you mean by that? Can you describe how you felt? What was the outcome of that situation?

**Table:** Table2. Graneheim and Lundman’s 5-step Content Analysis Approach

Step	Data analysis procedure
Transcription	Interviews were transcribed verbatim.
Meaning Units	Extracting key sentences and phrases relevant to the research topic
Abstraction	Summarizing meaning units and assigning appropriate conceptual codes.
Sorting the Codes	Grouping similar codes into conceptual categories and subcategories.
Category Formation	Choosing appropriate titles for categories

### Research Design

In this qualitative study, the researchers employed an inductive content analysis approach to understand the facilitators and barriers influencing professional role adaptation among NGNs during their first year of practice [24]. The study followed the COREQ (Consolidated Criteria for Reporting Qualitative Studies) reporting guidelines to ensure rigor and transparency in qualitative research [25].

### Recruitment and Participants

Participants in this study were purposefully selected from hospitals affiliated with Tehran University of Medical Sciences, located in central Iran. Initially, a list of NGNs with less than one year of clinical experience was obtained from the nursing managers of the selected hospitals. The nurses were then contacted via telephone and invited to the School of Nursing to participate in the study. Upon arrival, the researcher provided them with a participant information sheet and a consent form to be completed.

The inclusion criteria were being a newly graduated nurse with less than one year of clinical experience, aged 18 years or older, having direct experience with transitioning into a clinical nursing role, and being willing to participate voluntarily. Participants were selected purposively, and recruitment continued until data saturation was reached. Saturation was operationally defined as the point at which no new categories or subcategories emerged from the data. In this study, saturation was achieved after the 19th interview, as the subsequent two interviews yielded no additional insights, instead confirming the consistency of previously identified categories. The research team collaboratively reviewed and confirmed saturation through iterative analysis and validation of coding.

### Data Collection

Data collection began on October 23, 2023, and was completed on February 23, 2024. Data were gathered through semi-structured and in-depth interviews with NGNs. Each interview lasted between 50 and 80 minutes. The interview questions are presented in Table-1. These questions were developed based on a review of previous studies [19][26].

To ensure the validity of the interview guide, two pilot interviews were conducted with NGNs. Although the content of these pilot interviews was not included in the final data analysis, they played a critical role in refining the structure, sequencing, and phrasing of both the central and probing questions. Specifically, feedback from these pilot interviews led to reordering some questions to create a more natural flow, simplifying complex wording to improve clarity, and adding additional probes to elicit deeper responses regarding interpersonal and organizational challenges.

All subsequent interviews were audio-recorded with participants’ consent using a mobile device. The recordings were transcribed verbatim and reviewed multiple times to achieve a comprehensive understanding of the data. Nonverbal cues observed during the interviews were also incorporated into the transcripts to enhance contextual depth and interpretive accuracy.

### Data Analysis

Data were analyzed using conventional content analysis following the five-step approach proposed by Graneheim and Lundman [27][28]. The detailed data analysis process is outlined in Table-2. The first and second authors independently coded the interview transcripts. To evaluate inter-coder reliability and minimize subjective bias, 10% of the coded data were randomly selected and independently reviewed by both coders. Using the Intercoder Agreement function in MAXQDA version 20 (VERBI Software GmbH, Berlin, Germany), Cohen’s Kappa coefficient was calculated, yielding a value of 0.79, which indicates good agreement between coders. Any remaining discrepancies were discussed within the research team until consensus was reached. MAXQDA version 20 software was also used to manage and organize the data throughout the analysis.

### Trustworthiness and Rigor

Following data analysis, Lincoln and Guba’s four criteria were applied to ensure the study's rigor: credibility, transferability, dependability, and confirmability [29][30].

To enhance credibility, the researcher engaged in prolonged interaction with participants, establishing meaningful connections and spending sufficient time to gain a deep understanding of their experiences. Additionally, member checking was performed by returning to some participants after data analysis to validate the transcripts and ensure that the findings accurately reflected their views.

To ensure transferability, the study employed rich, detailed descriptions of the research context and provided thorough explanations of the data collection and analysis processes, allowing readers to determine the applicability of the findings to other settings.

Dependability was achieved through the transparent documentation of the research process and peer debriefing, which confirmed the consistency and reliability of the analytical steps.

Finally, confirmability was ensured by incorporating direct participant narratives to support the subcategories and minimize researcher bias.

### Ethical Considerations

This study was approved by the Joint Organizational Research Ethics Committee of the School of Nursing and Midwifery and the School of Rehabilitation at Tehran University of Medical Sciences. The project was registered under the code 981119900, and the study was granted ethical approval with the code IR.TUMS.FNM.REC.1402.010. The approval was officially issued on May 2, 2023. Before data collection, formal written permission was obtained from the management of the participating hospitals. Informed written consent was obtained from all participants after a clear explanation of the study objectives, methodology, and ethical considerations, including the confidentiality of their information and their right to withdraw at any time voluntarily. To ensure anonymity and data protection, all participant identifiers were replaced with numerical codes during data handling and reporting.

## Results

**Table:** Table3. Participants’ Characteristics

Participants ID	Gender	Age (years)	Marital status	Housing tenure status	Grade Point Average (GPA)	Working Shift	Work Experience (months)	Average working hours per month)	Hospital department
P1	Female	24	Single	owned	18.12	Rotational	2	209	Nephrology
P2	Male	24	Single	Rented	16.72	Rotational	4	280	General Surgery
P3	Female	23	Single	owned	17.50	Rotational	9	160	Cardiology
P4	Female	24	Single	owned	18.00	Rotational	7	210	Nephrology
P5	Female	24	Single	owned	16.50	Rotational	1	176	Orthopedic
P6	Female	25	Married	Rented	16.00	Rotational	1	186	ENT
P7	Female	25	Single	owned	18.69	Rotational	5	190	Women's Cancer Surgery
P8	Female	24	Single	owned	17.93	Rotational	8	200	Internal Medicine
P9	Female	24	Married	Rented	16.50	Rotational	11	180	Women’s Surgery
P10	Male	28	Single	Rented	16.01	Rotational	12	240	General Surgery
P11	Female	31	Married	owned	16.57	fix	7	140	Surgery
P12	Female	33	Single	Rented	18.24	Rotational	5	260	Surgery
P13	Female	32	Single	Rented	18.00	Rotational	10	190	Neurology
P14	Male	34	Married	owned	17.46	fix	12	175	Internal Medicine
P15	Female	24	Single	Rented	19.18	Rotational	6	180	Cancer
P16	Female	33	Single	Rented	13.94	Rotational	11	190	Dermatology
P17	Female	23	Single	Rented	16.50	Rotational	6	230	Surgery
P18	Female	24	Single	owned	18.50	Rotational	9	180	Women's Dermatology
P19	Female	24	Single	owned	17.30	Rotational	7	220	General Surgery
P20	Female	28	Single	Rented	19.00	fix	11	140	General
P21	Male	28	Married	owned	17.00	Rotational	11	160	General

**Table:** Table4. Facilitators and Barriers to NGNs’ Role Adaptation

**Main Category**	**Subcategory**	**Example Code**
	**Individual Facilitators **	Personal and personality traits, attention to assigned duties, individual effort, time management skills, previous student work experience, clinical knowledge and competence level, and interest in nursing.
**Facilitating Factors **	**Interpersonal factors **	Acceptance and support from colleagues, effective communication, and the quality of interpersonal interactions between staff and NGNs.
	**Organizational Facilitators **	Providing appropriate organizational training, supportive organizational management, and a positive organizational and societal culture.
	**Individual Barriers **	Stress and anxiety related to starting a new job, long commuting distances, sleep pattern disturbances, lack of motivation, insufficient scientific knowledge, unrealistic expectations, emotional sensitivity, and work-life conflict.
**Barriers** **Factors**	**Colleague-Related Barriers **	Lack of supportive coworkers, insufficient cooperation from colleagues, the absence of experienced staff to provide guidance, damaging or harmful behaviors from some team members, and poor communication within the workplace.
	**Organizational Barriers **	Excessive workload and patient assignments, a hostile work environment, frequent policy changes and bureaucratic regulations, shortage of equipment and resources, ineffective leadership from head nurses, insufficient income, and inadequate or non-specialized training programs.

Twenty-one NGNs participated in this study. Most participants were female (81%) and single (76%), aged 24 to 34. Additional demographic information is presented in Table-3. Comparison of participant responses based on demographic characteristics revealed that female NGNs reported facilitating factors in their role adaptation process more frequently than male NGNs. Additionally, participants aged 28 years and older tended to describe their adaptation experiences as smoother and more stable than those of their younger counterparts. These patterns emerged consistently across interviews, suggesting potential differences in adaptation trajectories based on gender and age.

### 1. Facilitating Factors

According to this study's findings, several factors contributed to facilitating the role adaptation of NGNs during their first year of employment in MSUs. These factors were identified at three levels: individual factors, interpersonal factors (related to colleagues), and organizational factors (Table-4). The interaction among these levels created a supportive environment that enabled successful adaptation to the professional nursing role.

### 1.1. Individual Facilitators

Based on the analysis of interview data, several individual-level factors were identified as facilitators in role adaptation among NGNs. These included personal and personality traits, attention to assigned duties, individual effort, time management skills, previous student work experience, clinical knowledge and competence level, and interest in nursing. Among these, "interest in nursing" emerged as a particularly influential factor. Participants who expressed a strong intrinsic motivation to become a nurse often described their professional role as a calling rather than just a job. This sense of purpose helped them navigate the challenges of clinical practice with greater resilience and perseverance. For example, one participant stated:

Participant 7: "Despite the stress and heavy workload, I love nursing. That love made it easier for me to stay and adapt."

Other participant quotes further support the relevance of individual-level facilitators:

Participant 11: "I believe the knowledge gained during nursing school is invaluable. That knowledge and skill assist a new nurse in adapting to clinical conditions."

Participant 19: "One of the things that helped me adapt was my student job. It made me stronger clinically."

Participant 18: "I believe the nurse herself is the first person who can help a newly graduated nurse."

### 1.2. Interpersonal Factors

According to the findings, positive interactions and relationships with colleagues were key facilitating factors in the role adaptation of NGNs during their first year of employment in MSUs. Within this subcategory, the main elements included acceptance and support from colleagues, effective communication, and the quality of interpersonal interactions between staff and NGNs. The following participant quotes illustrate these findings:

Participant 3: "Having proper communication with colleagues, even with support staff working on the ward, is important. When you’re friendly and communicate well with them, it becomes easier to get your work done."

Participant 15: "I believe what makes this period easier is when your colleagues accept you, embrace you, and take the time to teach you what you don’t know."

Participant 10: "Sometimes, a good colleague is even better than a good family."

### 1.3. Organizational Facilitators

The findings of this study revealed that key organizational factors significantly facilitated the role adaptation process for NGNs working in MSUs. These factors included providing appropriate organizational training, supportive organizational management, and a positive organizational and societal culture.

Participants emphasized that orientation and transition programs tailored to the needs of NGNs at the beginning of their employment were essential in helping them adapt to the new environment.

Participant 20: "Certainly, holding a realistic training program in the hospital based on the actual needs of NGNs can facilitate their adaptation to the new environment."

A supportive management structure and empathetic, experienced head nurses were also reported as primary facilitators of the organization. Participants noted that differences in managerial styles among head nurses significantly influenced their readiness and ability to adapt to the new environment.

Participant 11: "The trust shown by the head nurse and other nursing managers toward NGNs is one of the key factors that help with adaptation in the early stages of employment."

Some participants also referred to the broader organizational and societal culture. They believed that a positive and welcoming organizational culture in the hospital, combined with public support for the nursing profession, improved their outlook and eased the process of professional role adaptation.

Participant 8: "When the hospital culture is supportive and people respect nurses, it gives you more energy to stay and grow in your role."

These three levels of facilitators—individual, interpersonal, and organizational—did not operate in isolation but were interdependent and mutually reinforcing. For instance, organizational support, particularly through structured orientation programs, was perceived as most effective when combined with individual motivation and positive interpersonal relationships. Conversely, the absence of one level of support—such as weak managerial engagement—often diminished the effectiveness of the others, even among highly motivated nurses. Several participants also described how organizational barriers, particularly heavy workloads and a hostile work environment, intensified their stress and undermined their self-confidence, despite possessing strong clinical skills. In contrast, when organizational systems aligned with personal readiness and collegial support, participants reported smoother transitions and a greater sense of professional integration.

### 2. Barriers to Role Adaptation

According to this study's findings, NGNs encountered multiple barriers while adapting to the professional role within their first year of employment in MSUs. These hindering factors were identified at three primary levels: individual, colleague-related, and organizational (Table-4).

The complex and simultaneous interaction among these three barriers slowed the adaptation process, resulting in reduced quality of care, increased stress levels, and decreased job satisfaction among NGNs.

### 2.1. Individual Barriers

The findings of this study revealed that NGNs faced several individual-level challenges during their first year of clinical practice in MSUs that hindered their role adaptation. The most prominent individual barriers identified by participants included stress and anxiety related to starting a new job, long commuting distances, sleep pattern disturbances, lack of motivation, insufficient scientific knowledge, unrealistic expectations, emotional sensitivity, and work-life conflict. The following participant quotes illustrate these challenges:

Participant 3: "My commute from home to work is very long, making working conditions more difficult."

Participant 14: "In my opinion, the main barrier preventing nurses from adapting is the lack of motivation."

Some NGNs also noted that they had become passively accustomed to the existing conditions instead of actively adapting to their new role. This passive habituation prevented the resolution of more profound challenges.

Participant 7: "I still haven’t adapted to this situation. Adaptation doesn’t mean anything anymore — I’ve just gotten used to enduring the hardships

### 2.2. Colleague-related Barriers

The findings of this study indicated that one of the significant and influential barriers to role adaptation among NGNs in MSUs stemmed from challenges related to colleagues. Participants identified several issues in this category, including a lack of supportive coworkers, insufficient cooperation from colleagues, the absence of experienced staff to provide guidance, damaging or harmful behaviors from some team members, and poor communication within the workplace. The following participant quotes reflect these barriers:

Participant 11: "What prevents you from adapting well in the ward is the lack of cooperation from staff members, including the head nurse, attending physicians, and even support personnel."

Participant 15: "When we made a small mistake, some coworkers would tear down our character. It made us feel awful."

Participant 18: "I initially loved nursing and working in the hospital, but the inappropriate communication and mistreatment from some colleagues made me hate the profession".

### 2.3. Organizational Barriers

According to the results of this study, NGNs at the beginning of their professional careers in MSUs faced multiple organizational barriers in the adaptation process. These included excessive workload and patient assignments, a hostile work environment, frequent policy changes and bureaucratic regulations, a shortage of equipment and resources, ineffective leadership from head nurses, insufficient income, and inadequate or non-specialized training programs. The following participant quotes support these findings:

Participant 11: "The shifts were extremely heavy, and the number of patients was so high that working became unbearable for me."

Participant 15: "For me to adapt, there are much bigger problems, like the shortage of equipment, that haven’t been addressed yet. These make adaptation nearly impossible."

Lack of structured orientation programs at the beginning of employment and overreliance on routine-based rather than professional nursing training were also significant barriers to adaptation.

Participant 20: "When a nurse is confronted with a new situation without any preparation or training, it leads to stress, tension, and difficulty adapting".

## Discussion

This study aimed to identify and explore the facilitators and barriers to professional role adaptation among NGNs in MSUs. The findings revealed that the adaptation process is influenced by multidimensional factors operating at three levels: individual, interpersonal (colleague-related), and organizational. These levels interact with one another, collectively shaping the overall experience of professional transition for NGNs.

The complex and high-acuity nature of MSUs significantly influenced participants’ adaptation experiences. Frequent patient turnover, diverse clinical conditions, and constant time pressures were commonly cited as sources of stress and self-doubt, particularly in the absence of structured support systems or prior exposure to similar environments.

Findings of this study must be interpreted within the sociocultural and structural realities of the Iranian healthcare system. In Iran, the nursing profession is still influenced by entrenched cultural attitudes that tend to undervalue its role compared to medicine. Such societal perceptions can undermine NGNs’ professional identity and affect their treatment by patients, colleagues, and the wider public, thereby amplifying emotional strain during transition. Systemic challenges, including persistent staffing shortages, centralized management hierarchies, and limited orientation programs, further complicate the adaptation process. These contextual barriers help explain the prominent role of peer support and personal resilience observed among participants.

The findings of this study can also be interpreted through the lens of Duchscher’s Transition Shock Model, which describes the trajectory of newly graduated nurses as they move through the doing, being, and knowing stages of professional adaptation [31]. In the doing stage, NGNs in our study reported individual barriers such as stress, anxiety, unrealistic expectations, and disturbed sleep, reflecting the initial shock and uncertainty described by Duchscher. Progression into the beginning stage was characterized by reliance on interpersonal and organizational facilitators, including collegial support, mentorship, and structured orientation programs, which mirror Duchscher’s emphasis on the importance of socialization and guidance in mitigating transitional stress.

Finally, participants who were older or had greater resilience described experiences aligned with the knowing stage, where confidence, self-regulation, and a sense of professional identity emerged. This alignment between our categories and the stages of transition shock underscores the applicability of Duchscher’s model in explaining the complexities of NGNs’ role adaptation and highlights the need for targeted interventions across different phases of the transition process [32].

In the category of facilitating factors for role adaptation, the results of this study indicated that NGNs were able to adapt more effectively to the clinical environment by drawing on individual-level attributes such as personal effort, attention to assigned tasks, time management, prior student work experience, clinical competence, and a strong intrinsic interest in the nursing profession.

These findings are consistent with previous research by Kim and Shin (2020) and Tawash et al. (2024), which emphasized the importance of self-confidence, professional commitment, and personal motivation in facilitating role transition among NGNs [33][19].

However, this study extends the literature by highlighting the depth and interplay of these individual traits within the specific context of Iranian MSUs, where NGNs must often compensate for systemic shortcomings through enhanced personal initiative and resilience. While Murray et al. (2020) emphasized the value of educational interventions aimed at building psychological strengths, such as self-efficacy, the present study reveals how these traits become essential survival mechanisms in under-resourced, high-acuity environments. [34]. Based on these insights, it is recommended that nursing education in Iran—and similar healthcare settings—incorporate targeted content on resilience, self-leadership, and professional identity formation to better equip students for the transition to clinical roles.

At the interpersonal level, this study confirmed that acceptance, support, and constructive communication with colleagues were crucial facilitators of NGNs’ adaptation. These results align with studies by Hallaran et al. (2022), Kim and Shin (2020), and Faraz (2019), which emphasized the role of peer and supervisor support in promoting professional socialization and reducing transition-related anxiety [19][35][36]. However, our study adds context-specific nuance by showing that such interpersonal support often substitutes for formal orientation structures, which are frequently lacking in Iranian hospitals. This highlights the crucial role of team culture and peer dynamics in filling organizational gaps.

Supportive work environments, timely feedback, and collaborative interpersonal dynamics help reduce anxiety and feelings of isolation in NGNs, strengthen their sense of belonging, and increase retention [33][37]. Therefore, it is recommended that nursing managers and organizational decision-makers implement structured support systems, such as mentoring programs, regular feedback sessions, and initiatives to promote positive communication, to foster meaningful interaction and enhance belongingness among NGNs. These interventions can facilitate professional transition, reduce burnout, and improve nurse retention within healthcare systems.

At the organizational level, structured transition programs, initial orientation sessions, in-service training, and supportive management were identified as key contributors to successful role adaptation. These findings are aligned with studies by Alsalamah and Fawaz (2022), Kim and Shin (2020), Hallaran et al. (2022), and Tawash et al. (2024), all of which emphasize the effectiveness of structural interventions—such as transition programs and orientation sessions—in facilitating role adjustment [19][33][35][37]. In addition, structural empowerment in the workplace has been recognized as a significant driver of enhanced clinical competence, self-confidence, and job satisfaction [36][38].

Accordingly, it is recommended that healthcare policymakers design comprehensive and systematic evidence-based transition programs. These programs should incorporate psychological support, skills-based training, and continuous assessment of NGNs’ readiness to assume professional roles.

The findings of this study indicated that NGNs experienced a range of individual-level barriers, including a lack of motivation, stress, and anxiety associated with entering a new work environment, long commuting distances, inadequate sleep, unrealistic expectations, and conflicts with work-life balance. These results align with the concept of "reality shock" introduced by Duchscher (2008), which describes the psychological distress, anxiety, and confusion that NGNs often experience during the initial phase of professional entry [39].

Similarly, studies by Alsalamah and Fawaz (2022) and Kim and Shin (2020) identified fear and anxiety as significant barriers to a successful transition into the professional nursing role [19][37]. Kelly et al. (2021) also reported a direct correlation between high stress levels and increased turnover rates among NGNs [40].

A perceived lack of preparedness for clinical practice and insufficient hands-on experience were also found to undermine the role adaptation process—an issue also emphasized in the findings of Hallaran et al. (2022) and Tawash et al. (2024) [35][33]. These feelings can reduce job satisfaction and increase the risk of burnout, potentially leading to early attrition from the nursing profession. While these findings are consistent with existing literature, this study adds further nuance by illustrating how such stressors are compounded by contextual challenges specific to Iranian healthcare settings. For example, participants often linked emotional exhaustion to a perceived mismatch between academic preparation and real-world clinical expectations.

Given this evidence, it is recommended that nursing managers and healthcare policymakers prioritize developing and implementing structured psychological empowerment programs at the start of the transition period. These programs should include educational interventions, psychological counseling, and training in effective coping strategies to prevent emotional and behavioral distress and provide a supportive foundation for a healthy and successful professional transition.

At the interpersonal level, the findings indicated that unsupportive colleagues, destructive behaviors, lack of cooperation, and poor communication were among the most serious barriers to successful role adaptation in NGNs. Such conditions can erode professional self-confidence, foster a sense of rejection, and erode organizational belonging. Previous research—including studies by Alsalamah and Fawaz (2022), Hallaran et al. (2022), Kim and Shin (2020), and Faraz (2019)—has similarly highlighted the detrimental effects of insufficient social support and the prevalence of negative workplace cultures, particularly the presence of bullying behaviors, as significant obstacles to successful role adaptation [37][35][19][36].

However, this study offers a unique perspective by highlighting how, in the absence of formal support structures, interpersonal conflict and a lack of peer acceptance can become primary determinants of role disillusionment. This highlights the urgent need to embed interpersonal training and team-building interventions within orientation programs.

The absence of support during the critical early stages of transition may contribute to clinical errors, reduced quality of care, decreased job satisfaction, and increased turnover among NGNs [41].

Evidence shows that NGNs not accepted or supported by their colleagues and the organizational system tend to develop feelings of professional worthlessness and are significantly more vulnerable to burnout and eventual withdrawal from the profession [42]. Accordingly, it is recommended that nursing managers and healthcare policymakers develop and implement clear policies to foster positive workplace cultures, promote professional conduct, and address negative interpersonal behaviors. These efforts are essential for creating a safe, supportive, and collaborative environment for NGNs.

At the organizational level, this study's findings indicated that factors such as excessive workload, overcrowded units, lack of resources and equipment, weak clinical management, and insufficient ongoing training significantly disrupted the role adaptation process for NGNs. These findings are consistent with studies by Alsalamah and Fawaz (2022) and Kim and Shin (2020), which identified workload as one of the most critical barriers to a successful transition into the professional nursing role [19][37]. An imbalanced nurse-to-patient ratio, high-intensity shifts, and mandatory overtime severely restrict opportunities for learning, adjustment, and psychological recovery among NGNs, placing them under substantial pressure.

Faraz (2019) also highlighted the lack of adequate administrative support and inefficiencies in organizational structures as key challenges in the transition process [36]. These issues are particularly pronounced in the healthcare systems of developing countries, such as Iran, where the retention of qualified human resources is often not prioritized due to structural, economic, and managerial limitations. Under such conditions, increased emotional exhaustion, decreased job satisfaction, and increased turnover pose serious threats to the quality and continuity of care and clinical services.

Accordingly, it is recommended that healthcare authorities prioritize human resource retention policies, including reducing workload, improving nurse-to-patient ratios, providing adequate infrastructure and equipment, and offering continuous in-service training. In addition, reforming organizational structures by promoting transformational and accountable leadership may enhance job satisfaction and reduce turnover rates among NGNs.

Finally, a comparative analysis of participant characteristics suggested that demographic variables may influence the experience of professional role adaptation. Female participants more frequently emphasized facilitators, such as collegial acceptance, mentorship, and a supportive organizational culture, which were categorized under interpersonal and organizational aspects. In contrast, male participants more often described barriers such as professional isolation and limited guidance, which aligned with the category of colleague-related barriers. These differences may reflect gender-based variations in help-seeking behavior, emotional expressiveness, or access to social support, as has been noted in previous nursing literature [43].

Moreover, participants aged 28 years and older tended to describe a more stable adaptation process compared to their younger peers. Their accounts highlighted individual facilitators, such as emotional resilience, time management, and self-regulation. In contrast, younger nurses more frequently reported stress, unrealistic expectations, and professional vulnerability, which were categorized under individual and colleague-related barriers. Although the sample size does not permit statistical generalization, integrating these demographic trends with the study’s categories and subcategories suggests that age and gender may act as contextual moderators of role adaptation, underscoring the need for transition programs tailored to these differences.

The findings of this study have broad applications in nursing human resource management, professional education, and healthcare policy-making. At the operational level, nursing managers can utilize the evidence obtained to design more targeted support, training, and supervision programs tailored to the specific needs of NGNs during the initial stages of their clinical practice.

Strengthening facilitating factors, such as mentoring programs, skills-based training, and psychological support, and eliminating barriers, including excessive workload, poor communication, and hostile workplace cultures, can accelerate the adaptation process and help prevent burnout and early attrition.

Furthermore, the study’s findings can serve as a foundation for developing organizational policies and protocols to optimize the professional socialization process for NGNs. Such policies improve the quality of care in MSUs, contribute to human resource sustainability, and reduce costs associated with staff turnover.

This research has several limitations that should be considered when interpreting and generalizing the findings. First, the data were collected exclusively from hospitals affiliated with Tehran University of Medical Sciences. Therefore, generalizing the results to other healthcare settings with different organizational or geographical characteristics should be approached with caution and validated through further investigation. Second, the study participants were limited to NGNs working in MSUs. As a result, the findings may not fully reflect the experiences of nurses in other specialized departments such as intensive care units, emergency rooms, or operating theaters. Third, although the study employed purposive sampling and reached data saturation, as a qualitative study, it inherently relies on self-reported narratives, which may be influenced by recall bias or social desirability bias. Additionally, while the interviewer attempted to maintain neutrality, the possibility of interviewer bias in how questions were asked or how responses were interpreted cannot be entirely ruled out.

Furthermore, although demographic data were collected and specific trends (e.g., differences based on age or gender) were observed, the limited sample size restricted the ability to perform systematic comparative analysis. Future studies are encouraged to adopt larger or mixed-methods designs to explore how demographic characteristics such as gender, age, and work history influence the role adaptation process more comprehensively. Finally, as qualitative research inherently focuses on context-specific exploration, the participants' cultural, social, and organizational environment heavily influenced the findings. Thus, applying these results to different healthcare contexts—particularly outside Iran—requires cultural sensitivity and contextual adaptation.

## Conclusion

This qualitative study provides unique, context-specific insights into the professional role adaptation of NGNs in Iranian MSUs. These units, known for their complexity, high workload, and fast-paced environments, present distinct challenges, particularly in a healthcare system constrained by limited resources, hierarchical structures, and inadequate formal support mechanisms. Unlike studies conducted in Western contexts, this research highlights how cultural expectations, lack of structured orientation programs, and organizational inefficiencies interact with individual stressors, requiring NGNs to rely heavily on personal resilience and informal peer support. These findings contribute to a deeper understanding of how role adaptation unfolds within resource-constrained and culturally distinct settings, offering implications for both theory and practice.

Based on these insights, it is recommended that nursing managers and healthcare planners implement coordinated, context-specific, multi-level strategies tailored to the distinct demands of MSUs in Iran. These should include interventions aimed at strengthening individual competencies, promoting effective professional communication, and investing in organizational and educational infrastructure. Aligning support systems with the cultural and operational realities of high-acuity clinical settings can not only improve role adaptation outcomes and care quality but also reduce early attrition and enhance the long-term retention and professional integration of NGNs.

Future research should explore the long-term trajectories of role adaptation through longitudinal studies that follow NGNs beyond their first year of practice. In addition, mixed-methods approaches may help triangulate the subjective experiences of NGNs with objective indicators such as performance, retention rates, and patient outcomes. Further investigation into the impact of specific interventions—such as mentorship programs or simulation-based orientation—would also offer valuable evidence to guide policy and practice.

## Conflict of Interest

The authors declare that they have no known competing financial interests or personal relationships that could have appeared to influence the work reported in this paper.

## AI Disclosure Statement

During the preparation of this manuscript, the authors used ChatGPT, OpenAI company for language editing, grammar improvement, and liboberry.com for reference management. After its use, the authors thoroughly reviewed, verified, and revised all AI-assisted content to ensure accuracy and originality. The authors take full responsibility for the integrity and final content of the published article.

## References

[R1] Wang J, Xu Y, Zhang W, Guo Z, Zhang W, Zhang Y (2024). Transition status and influencing factors of newly graduated
nurses: a descriptive survey design. Nurs Educ Pract.

[R2] Woodward KF, Willgerodt M (2022). A systematic review of registered nurse turnover and retention in
the United States. Nurs Outlook.

[R3] Sokhanvar M, Kakemam E, Chegini Z, Sarbakhsh P (2018). Hospital nurses' job security and turnover intention and
factors contributing to their turnover intention: A crossSectional study. Nurs Midwifery Stud.

[R4] Kim Y, Kim HY (2021). Retention rates and the associated risk factors of turnover among
newly hired nurses at South Korean hospitals: A retrospective cohort study. Int J Environ Res Public Health.

[R5] Moon SH, Jeong HW, Jung US (2024). Exploring the impact of the mentoring new nurses for transition
and empowerment program led by clinical nurse educators in South Korea: a
mixedmethods study. Nurse Educ Today.

[R6] Park H, Yu S (2019). Effective policies for eliminating nursing workforce shortages: A
systematic review. Health Policy Technol.

[R7] Murray M, Sundin D, Cope V (2018). New graduate registered nurses’ knowledge of patient safety and
practice: A literature review. J Clin Nurs.

[R8] Li Y, Chi K, Li W, Sun X, Li Y (2023). Relationship between transition shock and humanistic practice
ability among Chinese newly graduated nurses: mediating effect of
organizational socialization. Nurs Educ Pract.

[R9] Aghaei N, Babamohamadi H, Asgari MR, DehghanNayeri N (2021). Barriers to and facilitators of nursing students’ adjustment to
internship: A qualitative content analysis. Nurse Educ Today.

[R10] JafarianAmiri SR, Zabihi A, Qalehsari MQ (2020). The challenges of supporting nursing students in clinical
education. J Educ Health Promot.

[R11] Callis AMB (2020). Application of the Roy Adaptation Theory to a care program for
nurses. Appl Nurs Res.

[R12] Kaihlanen AM, Haavisto E, Strandell‐Laine C, Salminen L (2018). Facilitating the transition from a nursing student to a
Registered Nurse in the final clinical practicum: a scoping literature
review. Scand J Caring Sci.

[R13] Kramer M (1975).

[R14] Duchscher JEB (2009). Transition shock: The initial stage of role adaptation for newly
graduated registered nurses. J Adv Nurs.

[R15] Phillips C, Esterman A, Kenny A (2015). The theory of organisational socialisation and its potential for
improving transition experiences for new graduate nurses. Nurse Educ Today.

[R16] Wong SWJ, Che WSW, Cheng MTC, Cheung CK, Cheung TYJ, Lee KY (2018). Challenges of fresh nursing graduates during their transition
period. J Nurs Educ Pract.

[R17] Cao X, Li J, Gong S (2021). The relationships of both transition shock, empathy, resilience
and coping strategies with professional quality of life in newly graduated
nurses. BMC Nurs.

[R18] Baharum H, Ismail A, McKenna L, Mohamed Z, Ibrahim R, Hassan NH (2023). Success factors in adaptation of newly graduated nurses: a
scoping review. BMC Nurs.

[R19] Kim JH, Shin HS (2020). Exploring barriers and facilitators for successful transition in
new graduate nurses: A mixed methods study. J Prof Nurs.

[R20] Graf AC, Jacob E, Twigg D, Nattabi B (2020). Contemporary nursing graduates’ transition to practice: A
critical review of transition models. J Clin Nurs.

[R21] Yıldız CÇ, Ergün Y (2020). Transition experiences of newly graduated nurses. Clin Exp Health Sci.

[R22] Aydogan Y, Ulupinar S (2020). Determining the learning needs of new graduated nurses working in
inpatient care institutions. Nurse Educ Today.

[R23] Hawkins N, Jeong S, Smith T (2019). New graduate registered nurses’ exposure to negative workplace
behaviour in the acute care setting: An integrative review. Int J Nurs Stud.

[R24] Kyngäs H (2019).

[R25] Tong A, Sainsbury P, Craig J (2007). Consolidated criteria for reporting qualitative research (COREQ):
a 32item checklist for interviews and focus groups. Int J Qual Health Care.

[R26] Woo MWJ, Newman SA (2020). The experience of transition from nursing students to newly
graduated registered nurses in Singapore. Int J Nurs Sci.

[R27] Graneheim UH, Lundman B (2004). Qualitative content analysis in nursing research: concepts,
procedures and measures to achieve trustworthiness. Nurse Educ Today.

[R28] Graneheim UH, Lindgren BM, Lundman B (2017). Methodological challenges in qualitative content analysis: A
discussion paper. Nurse Educ Today.

[R29] Guba EG, Lincoln YS (1982). Epistemological and methodological bases of naturalistic
inquiry. Ectj.

[R30] Elo S, Kääriäinen M, Kanste O, Pölkki T, Utriainen K, Kyngäs H (2014). Qualitative content analysis: A focus on trustworthiness. SAGE open.

[R31] Duchscher JB (2008). A process of becoming: The stages of new nursing graduate
professional role transition. The Journal of Continuing Education in Nursing.

[R32] Duchscher JB, Windey M (2018). Stages of transition and transition shock. Journal for Nurses in Professional Development.

[R33] Tawash E, Cowman S, Anwar M (2024). New graduate nurses’ readiness for practice, transition and
integration into the workplace: a longitudinal study with mixed methods
research. Nurs Educ Pract.

[R34] Murray M, Sundin D, Cope V (2020). Supporting new graduate registered nurse transition for safety: A
literature review update. Collegian.

[R35] Hallaran AJ, Edge DS, Almost J, Tregunno D (2023). New nurses’ perceptions on transition to practice: A thematic
analysis. Can J Nurs Res.

[R36] Faraz A (2019). Facilitators and barriers to the novice nurse practitioner
workforce transition in primary care. Journal of the American Association of Nurse Practitioners.

[R37] Alsalamah Y, Fawaz M (2023). Exploring facilitators and barriers for successful transition
among new Saudi graduate nurses: A qualitative study. Nurs Open.

[R38] Reebals C, Wood T, Markaki A (2022). Transition to practice for new nurse graduates: Barriers and
mitigating strategies. West J Nurs Res.

[R39] Duchscher JB (2008). A process of becoming: The stages of new nursing graduate
professional role transition. J Contin Educ Nurs.

[R40] Kelly LA, Gee PM, Butler RJ (2021). Impact of nurse burnout on organizational and position turnover. Nurs Outlook.

[R41] Jeffery J, Rogers S, Redley B, Searby A (2023). Nurse manager support of graduate nurse development of work
readiness: an integrative review. J Clin Nurs.

[R42] Senek M, Robertson S, Ryan T, King R, Wood E, Taylor B (2020). Determinants of nurse job dissatisfactionfindings from a
crosssectional survey analysis in the UK. BMC nurs.

[R43] Prosen M (2022). Nursing students’ perception of genderdefined roles in nursing: a
qualitative descriptive study. BMC Nurs.

